# COVID-19 and the ongoing emergency

**DOI:** 10.17843/rpmesp.2022.391.11121

**Published:** 2022-03-31

**Authors:** Lely Solari

**Affiliations:** 1 Instituto Nacional de Salud. Lima, Perú. Instituto Nacional de Salud Lima Peru

Two years have passed since the first patient with SARS-CoV-2 infection was diagnosed in Peru, since then more than 3.5 million cases and 212,000 deaths ^1^ have shown us that our health system, due to decades of neglect, poor financing and very slow management of infrastructure and equipment, was already chronically ill and in need of emergency care. At the beginning of the pandemic, there was a strong consensus on the need to maximize effort and allocate the necessary budget to the recovery of the health system, so that it could meet minimum standards, not of the first world countries, but at least of those in the same region ^2^.

However, at this stage of the pandemic, the recovery of the health system is considered increasingly less important, despite the persistent circulation of variants such as Omicron and in particular of its sub-lineages such as BA.2, which is more transmissible than its predecessors. Thus, currently, as we move to a “new normal” state in our society, the precarious situation of our health system is no longer a priority and it is once again functioning as it has historically done: according to the user’s resources.

The most frustrating aspect is that the actions needed to improve this situation are well known. Adequate management, setting realistic goals and deadlines to meet priorities defined by consensus, requires adequately trained and experienced managers. But working for the government is not attractive, neither economically nor professionally. Therefore, it is necessary to train and recruit managers, as was done some years ago, through incentive programs, such as those promoted by SERVIR. The establishment of meritocracy in the public recruitment selection process of key positions for technical personnel and directors of health facilities has been an issue postponed for years that should be resumed as soon as possible. In addition, there should be mechanisms to prevent administrative errors without increasing bureaucratic procedures. Penalizing civil servants for system failures leads to the paralysis of public administration and an increase of unnecessary procedures.

Another key element is the training of decision-makers in the field of evidence-based health policies, implementing procedures so that their actions are transparent and accountable. This would reduce the number of situations in which public policies deviate from scientific evidence, and if they do occur, there would be a mechanism to explicitly justify the motive. Interaction with business, when necessary, should be transparent.

Having a solid technical management team is not enough to ensure good public services. Restoring the credibility of public institutions, particularly health authorities, should also be important. There are very interesting studies that globally examine the determinants of success of pandemic management strategies, some of which found that the credibility of the authorities has an impact on the population’s adherence to regulations and on mortality caused by the pandemic ^3^. In other words, no matter how much our managers succeed in obtaining all the vaccines required to provide adequate coverage to our population, the expected results will not be achieved if people do not go to get them because of distrust, either in the vaccines themselves or in the people who promote them ^4^.

Recovering confidence in the government, regardless of the government in office, is a difficult but essential task. And the only way for the government to regain trust is by offering services that have a real and positive impact on citizens. The pandemic has made it clear that public services do not even come close to reaching an important sector of the population in our country, and other sectors are reached partially. It is not only about health services, basic services such as access to water and education are still lacking for many Peruvians, if solving this lack of state is not an emergency, what is?.

In addition, it is essential to regain faith that as Peruvians, we can come together, working as a team to ensure that all citizens have their basic needs covered. If a society is composed, apart from its rulers, by its citizens and social leaders, by academia, business, political parties, the media and other actors, we need each one to fulfill their role in favor of the society that gives them that role. We must learn from the experience of the pandemic, and prioritize those most in need. Because of scarcity, the most vulnerable population had to be prioritized to receive vaccination, but several other groups advocated for themselves to be vaccinated first: this cannot happen again. Solving the urgent and meeting the minimum for all must be the common goal of society as a whole.

For our part, we in the team of the Revista Peruana de Medicina Experimental y Salud Pública (Peruvian Journal of Experimental Medicine and Public Health) recognize that progress was made during the pandemic, but we must not get used again to the already chronic emergency of our health system and the lack of coverage of the basic needs of certain sectors of our population. From this small but significant space, and honoring the legacy of Dr. Zuño Burstein, we will continue to advocate for the dissemination of scientific information that contributes to provide solutions to urgent matters. We believe that science is an important instrument for us to achieve a better organized and fairer health system and society.
