# Supplementary material for: COVID-19 and the ongoing emergency
Source: Rev Peru Med Exp Salud Publica. 2022 Mar 31;39(1):4–5. doi: 10.17843/rpmesp.2022.391.11121 (PMC11397766; doi:10.17843/rpmesp.2022.391.11121)
Supplement: Translation

# Translation: COVID-19 y la emergencia que sigue vigente

**Authors:** Lely Solari

**Affiliations:** 1 Instituto Nacional de Salud. Lima, Perú.

Han pasado dos años desde que se diagnosticó el primer paciente con infección por SARS-CoV-2 en el Perú, desde entonces más de 3 millones y medio de casos y 212 000 fallecidos^ (^ ^1^ nos evidenciaron que, nuestro sistema de salud, con décadas de abandono, pobre financiamiento y muy lenta gestión de su infraestructura y equipamiento, era ya un enfermo crónico que necesitaba atención de emergencia. Al inicio de la pandemia, existía un fuerte consenso sobre a la necesidad de hacer el máximo esfuerzo y asignar el presupuesto necesario a la recuperación del sistema sanitario, de tal manera que pudiera cumplir con los estándares mínimos, no de los países del primer mundo, pero por lo menos de los de la región ^2^.

Sin embargo, en esta etapa de la pandemia, pese a la persistente circulación de variantes como la Omicron y en particular de sus sub-linajes como la BA.2, más transmisible que sus antecesoras, se le da cada vez menos importancia a la recuperación del sistema de salud. Es así como, en la actualidad, al virar a la “nueva normalidad” del funcionamiento de nuestra sociedad, la situación precaria de nuestro sistema sanitario deja ya de ser una prioridad y vuelve a funcionar como históricamente lo ha hecho: de acuerdo a los recursos del usuario.

Lo más frustrante es que las acciones que se necesitan para mejorar esta situación son conocidas. Para tener una adecuada gestión, que establezca metas y plazos realistas para cumplir con prioridades definidas mediante consenso, se requieren gestores adecuadamente capacitados y con experiencia. Pero trabajar para el estado no es atractivo, ni económica ni profesionalmente. Entonces resulta necesaria la formación y captación de gerentes, como se hacía hace algunos años, a través de programas de incentivos, como los que impulsaba SERVIR. El concurso público para la selección de puestos técnicos clave y directores de los establecimientos de salud para instituir la meritocracia en el sistema, es un tema que se ha postergado por años y debe de retomarse a la brevedad. Además, deben de existir mecanismos para prevenir errores administrativos sin incrementar los trámites burocráticos. Penalizar al funcionario por fallas del sistema induce a la parálisis de la gestión pública y el fomento de tramites innecesarios.

Otro elemento clave es la capacitación de los decisores en el ámbito de políticas en salud basadas en evidencia, implementando procedimientos para que sus disposiciones sean transparentadas y exista rendición de cuentas. De esta manera disminuyen las situaciones en las que las políticas públicas se alejan de la evidencia científica, y si ocurren, existiría un mecanismo por el cual se sustente explícitamente el motivo. La interacción con la empresa, cuando es necesaria, debe de ser transparente.

Tener un equipo técnico sólido en la gestión no es suficiente para asegurar buenos servicios públicos. Se debe de dar importancia también a recuperar la credibilidad de las instituciones públicas y en particular, de las autoridades sanitarias. Existen estudios muy interesantes que examinan a nivel global los determinantes del éxito en el manejo de la pandemia, algunos de los cuales han encontrado que la credibilidad en las autoridades impacta en la adherencia por parte de la población a las normas que estas imparten y en la mortalidad producida por la pandemia en sí ^3^. Es decir, por más que nuestros gestores logren conseguir todas las vacunas que se requieren para dar adecuada cobertura a nuestra población, si la gente no acude a colocárselas por desconfianza, ya sea en las propias vacunas como en las personas que las impulsan, no se van a alcanzar los resultados esperados ^4^.

Recuperar la confianza perdida en el estado, de manera independiente de cuál es el gobierno de turno, es una tarea difícil, pero imprescindible. Y la única manera de que el estado genere confianza es ofreciendo servicios que tengan impacto real y positivo para los ciudadanos. La pandemia ha dejado en evidencia que hay un importante sector de la población en el país a la que los servicios públicos ni siquiera se acercan, y otros a los que llegan de manera insuficiente. No sólo se trata de servicios de salud, servicios tan básicos como el agua, y la educación todavía le hacen falta a muchos peruanos, si solucionar esa falta de estado no es una emergencia, ¿qué lo es?

Además, es imprescindible recuperar la fe en que como peruanos, podemos ponernos de acuerdo, trabajando en equipo para lograr que todos los ciudadanos tengamos lo básico. Si una sociedad está compuesta, aparte de sus gobernantes, por sus ciudadanos y líderes sociales, por la academia, la empresa, los partidos políticos, los medios de comunicación y demás actores, necesitamos que cada uno cumpla su rol en pro de la sociedad que les otorga ese papel. Debemos aprender de la experiencia de la pandemia, y priorizar a los más necesitados. Cuando se tenía que priorizar las vacunas para los más vulnerables porque eran escasas, diversos grupos concentraban sus energías en abogar por que a ellos les llegaran antes: eso no puede volver a ocurrir. Solucionar lo urgente y cumplir lo mínimo para todos debe ser la meta común de la sociedad en su conjunto.

Por nuestra parte, en el equipo de la Revista Peruana de Medicina Experimental y Salud Pública reconocemos los avances obtenidos durante la pandemia, pero no debemos acostumbrarnos nuevamente a la ya crónica emergencia de nuestro sistema de salud y a la falta de cobertura de las necesidades básicas de ciertos sectores de nuestra población. Desde este pequeño pero significativo espacio, y honrando el legado del Dr. Zuño Burstein, seguiremos abogando por difundir la información científica que contribuya a dar solución a lo urgente. Creemos que la ciencia es un importante instrumento para que logremos un sistema sanitario y una sociedad mejor organizados y más justos.
